# AFLP Genome Scan to Detect Genetic Structure and Candidate Loci under Selection for Local Adaptation of the Invasive Weed *Mikania micrantha*

**DOI:** 10.1371/journal.pone.0041310

**Published:** 2012-07-19

**Authors:** Ting Wang, Guopei Chen, Qijie Zan, Chunbo Wang, Ying-juan Su

**Affiliations:** 1 CAS Key Laboratory of Plant Germplasm Enhancement and Specialty Agriculture, Wuhan Botanical Garden, Chinese Academy of Sciences, Wuhan, China; 2 State Key Laboratory of Biocontrol, School of Life Sciences, Sun Yat-sen University, Guangzhou, China; 3 Shenzhen Wildlife Rescue and Rehabilitation Center, Shenzhen, China; University of Oxford, United Kingdom

## Abstract

Why some species become successful invaders is an important issue in invasive biology. However, limited genomic resources make it very difficult for identifying candidate genes involved in invasiveness. *Mikania micrantha* H.B.K. (Asteraceae), one of the world's most invasive weeds, has adapted rapidly in response to novel environments since its introduction to southern China. In its genome, we expect to find outlier loci under selection for local adaptation, critical to dissecting the molecular mechanisms of invasiveness. An explorative amplified fragment length polymorphism (AFLP) genome scan was used to detect candidate loci under selection in 28 *M. micrantha* populations across its entire introduced range in southern China. We also estimated population genetic parameters, bottleneck signatures, and linkage disequilibrium. In binary characters, such as presence or absence of AFLP bands, if all four character combinations are present, it is referred to as a character incompatibility. Since character incompatibility is deemed to be rare in populations with extensive asexual reproduction, a character incompatibility analysis was also performed in order to infer the predominant mating system in the introduced *M. micrantha* populations. Out of 483 AFLP loci examined using stringent significance criteria, 14 highly credible outlier loci were identified by Dfdist and Bayescan. Moreover, remarkable genetic variation, multiple introductions, substantial bottlenecks and character compatibility were found to occur in *M. micrantha*. Thus local adaptation at the genome level indeed exists in *M. micrantha*, and may represent a major evolutionary mechanism of successful invasion. Interactions between genetic diversity, multiple introductions, and reproductive modes contribute to increase the capacity of adaptive evolution.

## Introduction

Why some species become successful invaders is an important issue in invasive biology. When species are introduced into a new region, they face two fates. Some species quickly go extinct, whereas others persist and finally become highly competitive invaders, posing a serious threat to native diversity and ecosystems [1], [2]. Successful invasions involve three major phases: introduction, naturalization, and invasion [3], [4]. In the initial introduction phase, invasive species often contain low levels of genetic diversity due to bottleneck and founder effects [5]–[8]. Then, invaders produce pre-adapted genotypes in response to the abrupt environmental changes during naturalization [9]–[12]. Finally, exotic species become broadly invasive in the extended period [9], [13]. The rapid population expansion of invaders is expected to promote adaptive evolution, since it has been shown that the rapidly increasing population size is conducive to withstanding (and responding to) strong directional selection [14], [15]. A substantial time lag is involved during the transition from introduction via naturalization to invasion [4]. The occurrence of a lag phase allows populations to adapt to new environmental factors such as ecological niche, temperature, precipitation, soils, frost, and wind speed or growing season length [11], [12], [16]–[18]. On the other hand, neutral or deleterious alleles, which become favored in new ecological contexts, will contribute to adaptive changes of invasive populations [19]. These changes may increase the survival rate of invasive species [12], making them become gradually dominant in the introduced range [20]. Therefore, pre-adaptation to novel environments is often counted as a premise for successful invasion [3], [4], [21].

Genomic scans are useful to identify potential adaptive loci under selection at the genomic level [22], [23]. All loci across the genome are anticipated to possess similar demography and neutral evolution history of populations, including genetic drift and gene dispersal [24]. If variation of a locus is beyond the genomic pattern with an unusual frame of higher genetic differentiation, it is deemed an “outlier locus” under natural selection [24], [25]. The outlier locus can be identified explicitly in the genes under selection and also in neutral flanking regions due to hitchhiking effects [26], [27]. In model organisms for which whole genomic information is available, it is easy to track the “outlier locus” under selection [28]. However, for non-model organism such as invasive species, it becomes difficult to identify candidate genes and pinpoint the evolutionary and genetic factors involved in invasiveness because of restricted genomic resources [29], [30]. New methods, especially those based on the polymerase chain reaction (PCR) to obtain amplified polymorphisms, have frequently been used to scan genomes in non-model organisms [28], [31]. Among these, amplified fragment length polymorphisms (AFLPs) are reported to be the most efficient approach to identify candidate genomic regions under selection (candidate loci or outlier loci) [22], [23], [31]–[37]. They can provide several hundred random loci scattered throughout the genome at less cost [37]. So far, the most commonly used analysis approaches for AFLP genome scans are Dfdist and the hierarchical Bayesian method (Bayescan). Dfdist, which was originally developed by Beaumont & Nichols [38], employs a classical Wright's island model to generate the expected neutral distribution of *F*_ST_ estimates [39]–[41]. In contrast, Bayescan relies on a logistic regression model [42], representing an extended method devised by Beaumont & Balding [43]. To decrease false positives, Dfdist mainly depends on the trimmed mean of the empirical *F*_ST_ distribution for the simulation [44], whereas Bayescan uses a likelihood ratio test to evaluate the most likely of two alternative models, one that includes the effect of selection and another that excludes it [41], [42]. Bayescan has been suggested to be more efficient at detecting high selective loci with low false positives [45].

*Mikania micrantha* H. B. K. (Asteraceae) is one of the top ten worst weeds in the world [46]. It is a many-branched climbing perennial vine native to tropical Central and South America where it is a weed of minor importance [46]–[48]. *M. micrantha* was deliberately introduced into Asia as early as 1900s [49]. In humid sub-tropical China, *M. micrantha* is subjected to new selective pressures, including such abiotic and biotic stresses as new climate, soils, pathogens, herbivores, pollinators, and competitors [50]. By adaptive evolutionary changes, *M. micrantha* survived and reproduced in new environments using strikingly different strategies from those employed in its original niche [19]. It demonstrated the ability to outcompete native plants in utilizing such limited resources as soil nutrients and sunlight and releasing phytotoxic compounds to inhibit growth of neighboring plants [51]–[54]. Once established, *M. micrantha* became a dominant plant across most regions, influencing ecosystems, biological diversity, and natural communities [50]. By the late 1980s and early 1990s, it had spread extensively in southern China, colonizing agricultural land, orchards, nurseries, lawns, mangroves, secondary forests, scrubland, waste ground, ponds, and seashore [55], [56]. Unlike in its native range, in southern China *M. micrantha* grows on dry soils as well as shady sites [56]. Its favorable growth conditions have changed to an average annual temperature higher than 21°C and soil moisture content over 15% [52]. Since spreading at accelerating rates, it is reasonable to postulate that adaptive evolution provides a key mechanism allowing the success of *M. micrantha* in new environments [31], [57]. Such adaptive processes may leave specific signatures in the genome of *M. micrantha* [36]. Thus we expected to detect the “outlier locus” signature of such local adaptations under selection at the genomic level. It is particular important to find the “outlier locus” associated with the local adaptation because, firstly, the information is critical to understanding the introduction history and genetic consequences of introduced populations of *M. micrantha* [2], [30]. Second, it may provide fresh insights into the evolutionary potential of *M. micrantha* populations, which is helpful to predict their adaptability in response to management practices [9], [58]. Finally, the locus will offer a prime candidate for functional surveys targeting the linked gene and dissecting the molecular mechanism of invasiveness [36].

**Figure 1:**
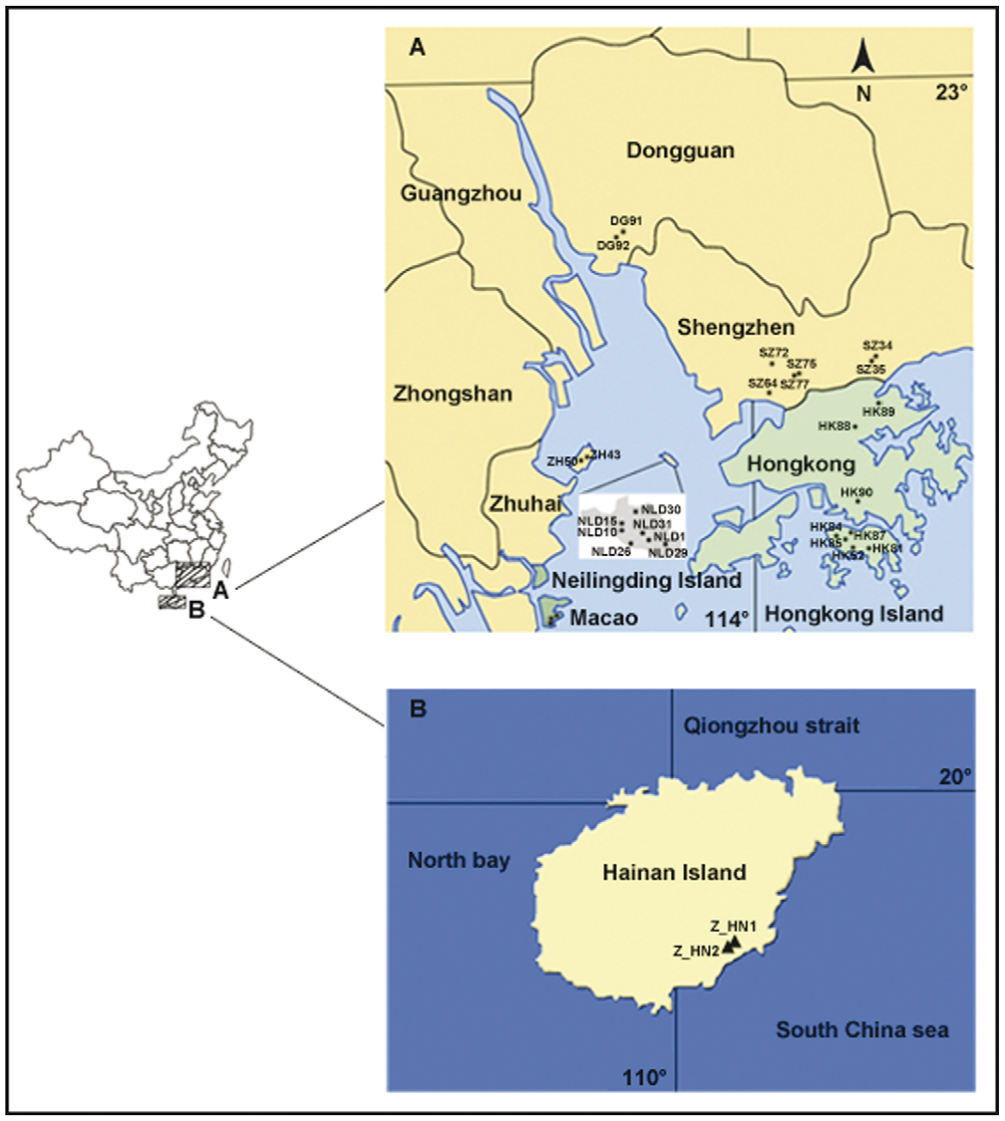
Map of sampled populations. (A) *Mikania micrantha*. (B) *Mikania cordata*.

To test our prediction that outlier loci can be found in southern China populations of *M. micrantha* (Figure 1; Table 1) and to detect genetic structure, in the present study we performed a genome scan analysis based on a large number of AFLP polymorphisms. The goals of the study were (1) to identify candidate loci under selection for local adaptation in *M. micrantha* and (2) to understand contributions of interactions between genetic diversity, reproductive modes, bottlenecks, and multiple introductions to the adaptive evolution of *M. micrantha*.

**Table 1:** Locations of *Mikania micrantha* and *Mikania cordata* populations surveyed in this study.

Species	Region	Population	Location	Latitude	Longitude	Sample size	Altitude (m)
*M. micrantha*	Hong Kong	HK81	Hong Kong Island, Stubbs Road, in shrubs	22° 16′ 08″ N	114° 10′ 49″ E	15	74
		HK82	Hong Kong Island, Barker Road, in tussock	22° 16′ 09″ N	114° 09′ 50″ E	13	300
		HK84	Hong Kong Island, Mount Gough, in tussock	22° 16′ 05″ N	114° 09′ 43″ E	16	315
		HK85	Hong Kong Island, Victoria Peak, in tussock	22° 16′ 30″ N	114° 08′ 57″ E	15	503
		HK87	Hong Kong Zoological and Botanical Gardens, under bamboo forest	22° 16′55″N	114° 09′23″ E	12	372
		HK88	New Territories, Hok Tau, in tussock	22° 29′ 53″ N	114° 10′ 49″ E	15	46
		HK89	New Territories, Luk Keng, Pat Sin Leng Country Park, rivulet-side	22° 31′ 26″ N	114° 12′ 57″ E	12	8
		HK90	Kowloon, Hong Kong Baptist University, slope	22° 20′ 07″ N	114° 10′ 57″ E	19	25
	Macao	MA101	Hác Sá Beach, wasteland	22° 07′ 08″ N	113° 34′ 04″ E	15	3
		MA105	Hác Sá Village, roadside	22° 07′ 02″ N	113° 34′ 03″ E	15	1
		MA106	Hác Sá Beach, building site	22° 07′16″N	113°34′10″E	14	8
	Shenzhen	SZ34	Fairy Lake Botanical Garden, Liang Yi Ting, in tussock	22° 34′ 49″ N	114° 10′ 08″ E	14	88
		SZ35	Fairy Lake Botanical Garden, Desert Plant Section, roadside	22°35′10″N	114°10′26″E	16	42
		SZ64	The office of Mangrove Natural Reserve, roadside	22° 31′ 58″ N	114° 00′ 01″ E	11	19
		SZ72	Meilin Park, Huanshan Road, slope	22° 33′ 59″ N	114° 01′ 27″ E	16	83
		SZ75	Lotus Hill Park, the Kite Square, in tussock	22° 33′ 14″ N	114° 03′ 22″ E	14	38
		SZ77	Lotus Hill Park, under the Eucalypt forest	22° 33′ 25″ N	114° 02′ 57″ E	16	46
	Neilingding	NLD1	Management station, roadside	22° 23′ 48″ N	113° 49′ 09″ E	15	3
		NLD10	Dong Jiao Zui Wan, slope, in shrubs	22° 24′ 06″ N	113° 48′ 44″ E	14	51
		NLD15	Dong Jiao Zui Wan, slope, under Spiny date palms	22°24′21″N	113°48′42″E	17	145
		NLD26	Nan Wan, in shrubs	22° 23′ 41″ N	113° 48′ 52″ E	14	3
		NLD29	Dong Wan, ravine	22° 23′ 48″ N	113° 49′ 38″ E	15	15
		NLD30	Bei Wan Ma Guan, in shrubs	22° 25′ 12″ N	113° 47′ 17″ E	15	3
		NLD31	Management station East, ravine	22° 24′ 01″N	113° 49′ 59″ E	14	8
	Zhuhai	ZH43	Qi'ao Island, roadside	22° 24′ 37″ N	113° 38′ 38″ E	15	2
		ZH50	Qi'ao Island, No Jia Le, roadside	22° 24′ 03″ N	113° 37′ 40″ E	12	2
	Dongguan	DG91	Da Ling Shan forestry centre, Shan Zhu Wo	22° 51′ 51″ N	113° 46′ 21″ E	16	174
		DG92	Da Ling Shan forestry centre, Chang Keng Kou	22° 51′ 38″ N	113° 46′ 27″ E	15	114
*M. cordata*	Hainan Island	HN1	Xinglong, in arbors	18° 42′04″ N	110° 13′ 23″ E	12	49
		HN2	Xinglong, in tussock	18° 42′03″ N	110° 13′ 22″ E	5	44

## Results

### Population genetic variation

AFLP analysis of *M. micrantha* was optimized by determining which selective primer sets produced the clearest DNA fragments based on the eight primer pair combinations. The eight primer pairs were polymorphic in each population. A total of 483 clear polymorphic fragments were detected. The proportion of polymorphic loci within a population ranged from 10.57% to 73.47%, with an average value of 41.84% (Table 2). The average number of fragments per individual was 338.39, and 67.75 per primer combination.

**Table 2:** Estimates of genetic diversity, and test for linkage disequilibrium and character compatibility in populations of *Mikania micrantha*.

Population	Number of loci	Number of polymorphic loci	Percentage of polymorphic loci	Nei's gene diversity	*I_A_*	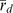	*IER*
HK81	426	313	0.7347	0.2932	7.5391**	0.0242**	0.079**
HK82	427	273	0.6393	0.2548	6.3634**	0.0234**	0.027**
HK84	387	125	0.323	0.1358	15.5087**	0.1252**	0.512**
HK85	426	188	0.4413	0.1792	10.7784**	0.0576**	0.405**
HK87	383	95	0.248	0.0961	3.0253**	0.0322**	0.076*
HK88	429	205	0.4779	0.1957	7.8618**	0.0385**	0.261**
HK89	446	243	0.5448	0.2101	0.2056	0.0009	0.011
HK90	413	203	0.4915	0.1977	6.3289**	0.0314**	0.065**
Hong Kong	474	442	0.9325	0.3302	6.5539**	0.0160**	0.01**
MA101	418	237	0.567	0.2187	4.1371**	0.0175**	0.052**
MA105	389	173	0.4447	0.1773	8.8737**	0.0516**	0.004
MA106	369	39	0.1057	0.0424	3.0656**	0.0807**	0.257*
Macao	442	318	0.7195	0.2664	19.7051**	0.0641**	0.068**
SZ34	435	176	0.4046	0.1638	17.5379**	0.1002**	0.405**
SZ35	386	85	0.2202	0.0928	2.1485**	0.02560**	0.011
SZ64	437	212	0.4851	0.1936	9.7626**	0.0463**	0.255**
SZ72	373	58	0.1555	0.07	7.8698**	0.1382**	0.101
SZ75	451	267	0.592	0.234	6.2308**	0.0234**	0.006
SZ77	433	243	0.5612	0.2265	22.1611**	0.0916**	0.206**
Shenzhen	471	366	0.7771	0.287	7.2587**	0.2091**	0.031**
NLD1	426	196	0.4601	0.1863	20.7551**	0.1065**	0.346**
NLD10	381	52	0.1365	0.0547	3.8665**	0.0759**	−0.074
NLD15	430	180	0.4186	0.1599	12.4718**	0.0697**	0.216**
NLD26	390	59	0.1513	0.0639	6.7266**	0.1160**	0.096
NLD29	437	223	0.5103	0.2016	18.5328**	0.0835**	0.170**
NLD30	373	47	0.126	0.0545	5.4156**	0.1178**	−0.078
NLD31	410	141	0.3439	0.1402	33.0933**	0.2365**	−0.001
Neilingding	470	350	0.7447	0.2604	9.7616**	0.0303**	0.087**
ZH43	438	290	0.6621	0.2622	13.6221**	0.0472**	0.366**
ZH50	406	267	0.6576	0.2723	14.4875**	0.0545**	0.032*
Zhuhai	457	376	0.8228	0.3193	10.4551**	0.0281**	0.121**
DG91	438	184	0.4201	0.1655	13.6645**	0.0747**	0.420**
DG92	435	171	0.3931	0.1515	22.3371**	0.1315**	0.478**
Dongguan	458	241	0.5262	0.2028	6.7332**	0.0282**	0.180**
Total	483	483	1.0000	0.3376			

*I_A_*, index of association; 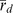, modified index of association; *IER*, incompatibility excess ratio.

* , *P*<0.05;

** , *P*<0.01.

At the regional level, either Hong Kong or Zhuhai was consistently found to maintain the highest level of variation according to all the genetic diversity statistics (Table 2). However, populations such as MA106, SZ72, NLD10, NLD26, and NLD30 growing in regions of Macao, Shenzhen, and Neilingding were observed to possess lower amounts of genetic variation than others in the same region (Table 2).

### Linkage disequilibrium and character compatibility

We estimated levels of linkage disequilibrium (LD) and character compatibility among the 483 AFLP loci. Each region was observed to possess significant LD and matrix compatibility obtained by testing the index of association (*I*_A_ and 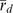) and the incompatibility excess ratio (IER) (Table 2). Likewise, significant LD and matrix compatibility were also detected at the population scale (Table 2).

### Allele frequency distribution and bottleneck signature test

Alleles were defined as rare when they occurred at a frequency of less than 0.05 in the examined populations [59]. In total, 22 rare alleles were identified at the species level. Across regions, 14, 12, and 9 rare alleles were detected in Hong Kong, Shenzhen, and Neilingding, respectively, whereas no rare alleles were found in Macao, Zhuhai, or Dongguan. In addition, no rare allele was detected within any populations at the population level.

Using the stepwise mutation model (SMM) and the infinite allele model (IAM), we identified bottleneck signatures in each population with a heterozygosity excess/deficiency ratio that significantly deviated from the expected ratio (1∶1) at mutation-drift equilibrium (Table 3, P<0.05). Moreover, the entire range of *M. micrantha* appeared subject to a significant bottleneck (Table 3).

**Table 3:** Genetic bottleneck of *Mikania micrantha* populations from six introduced regions in southern China.

	SMM		IAM	
Region	*H_e_*/*H_d_*	*P*	*H_e_*/*H_d_*	*P*
HK81	268/45	0.00000	268/45	0.00000
HK82	230/43	0.00000	230/43	0.00000
HK84	101/24	0.00000	102/23	0.00000
HK85	156/32	0.00000	156/32	0.00000
HK87	75/20	0.00000	75/20	0.00000
HK88	172/33	0.00000	172/33	0.00000
HK89	202/41	0.00000	202/41	0.00000
HK90	175/28	0.00000	175/28	0.00000
Hong Kong	277/165	0.00000	302/140	0.00000
MA101	177/60	0.00000	177/60	0.00000
MA105	138/35	0.00000	138/35	0.00000
MA106	30/9	0.00448	30/9	0.00007
Macao	199/119	0.00000	242/76	0.00000
SZ34	149/27	0.00000	149/27	0.00000
SZ35	72/13	0.00000	72/13	0.00000
SZ64	174/38	0.00000	174/38	0.00000
SZ72	52/6	0.00000	52/6	0.00000
SZ75	230/37	0.00000	230/37	0.00000
SZ77	212/31	0.00000	212/31	0.00000
Shenzhen	243/123	0.00000	289/77	0.00000
NLD1	171/25	0.00000	171/25	0.00000
NLD10	41/11	0.00032	41/11	0.00000
NLD15	149/31	0.00000	149/31	0.00000
NLD26	50/9	0.00000	50/9	0.00000
NLD29	186/37	0.00000	186/37	0.00000
NLD30	42/5	0.00000	42/5	0.00000
NLD31	119/22	0.00000	119/22	0.00000
Neilingding	222/128	0.00000	238/112	0.00000
ZH43	250/40	0.00000	250/40	0.00000
ZH50	221/46	0.00000	221/46	0.00000
Zhuhai	311/65	0.00000	323/53	0.00000
DG91	146/38	0.00000	146/38	0.00000
DG92	113/58	0.00000	115/56	0.00000
Dongguan	201/40	0.00000	214/27	0.00000
Total	309/174	0.00000	337/146	0.00000

*P* values are determined by a sign test under the stepwise mutation model (SMM) and the infinite allele model (IAM). *H_e_*/*H_d_*, the heterozygosity excess/deficiency ratio.

### Population genetic differentiation and relationship

The genetic differentiation *Φ*_ST_ measured by AMOVA analysis was 0.3335; 66.65% of the variation was partitioned within populations, 28.61% was attributed to differences among populations within regions, and only 4.74% of the variation was due to regional differences (all three hierarchical levels were significant with P<0.001) (Table 4). When individual pairs of populations were compared, of the 378 pairwise *Φ*_ST_ values, 373 were significant (P<0.001), whereas only five values derived from SZ64 vs. SZ75, SZ64 vs. SZ77, SZ64 vs. DG91, SZ75 vs. SZ77, and SZ75 vs. DG91 were not (P>0.05), highlighting remarkable differences among populations.

**Table 4:** Analysis of molecular variance (AMOVA) of 483 AFLP loci for 28 *Mikania micrantha* populations from six introduced regions in southern China.

	Variance components	Percentage of total variation	*P*	*Φ* statistics
Among regions	2.884	4.74	<0.001	*Φ_CT_* = 0.0474
Among populations within regions	17.404	28.61	<0.001	*Φ_SC_* = 0.3004
Within populations	40.538	66.65	<0.001	*Φ_ST_* = 0.3335

The *P*-value was calculated by a permutation procedure based on 1023 replicates.

The *f*-free model chosen based on the smallest mean DIC was more suitable than other models for the AFLP data set of the 28 populations. Therefore, *θ^B^* = 0.2927 (95% credible interval: 0.2772–0.3058) was determined to be an unbiased Bayesian estimate of all population genetic differentiation.

No significant associations between geographical distances and pairwise estimates of *θ^B^* were observed in the full AFLP data set (r = 0.0032, P = 0.452). A similar pattern was derived at the regional level. Hence no evidence of “isolation by distance” [60] was revealed by Mantel tests at either the regional or entire range level.

**Figure 2:**
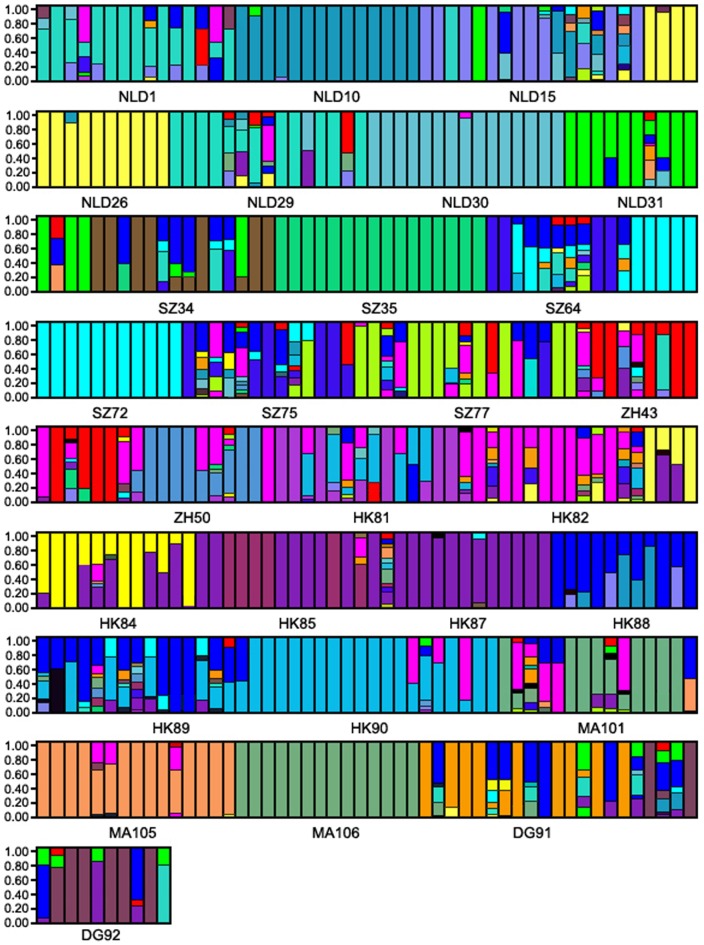
Bayesian assignment proportions for *K* = 28 clusters determined in STRUCTURE 2.3.3. Each vertical bar represents one individual.

The *ΔK* criterion of Evanno *et al.* [61] was applied to estimate the number of population clusters. The maximal value of *ΔK* was *K* = 24, indicating the need to divide the samples into 24 clusters. Bar plots showed varying extents of admixture among populations (Figure 2). Ten clusters including populations NLD10, NLD26, NLD30, NLD31, SZ35, SZ72, HK87, HK90, MA105, and MA106 were detected, although each population also contained a very few individuals from different regions. The substructure of the rest populations was weak and could only be resolved when information about the sampling locations was included. A mix of individuals from other clusters was often observed in each cluster. The results suggested that *M. micrantha* populations in southern China maintained a relatively weak genetic structure.

**Figure 3:**
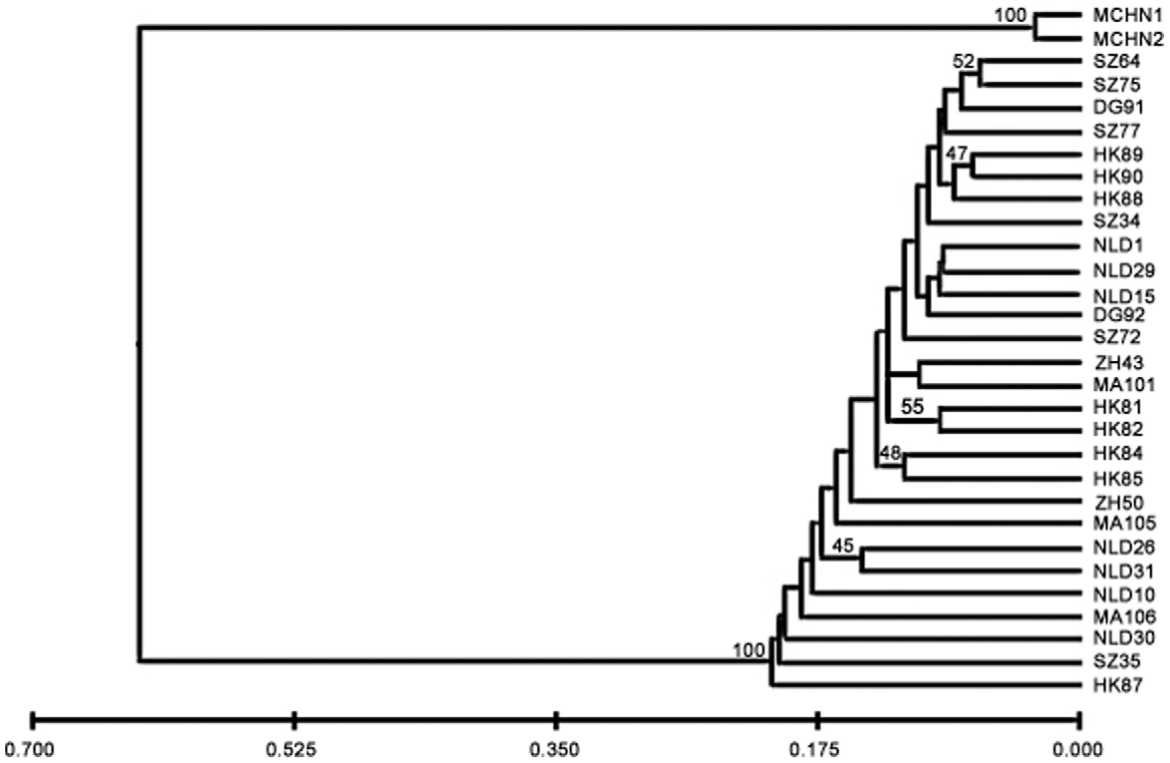
UPGMA dendrogram derived from AFLP data by Nei's [**105**] unbiased genetic distances. It shows the relationships among 28 examined populations of *Mikania micrantha*. Populations of *Mikania cordata* are used as the root. Numbers above branches indicate bootstrap values (% of 1000 replicates). Only values larger than 40% are displayed. Branch lengths are proportional to genetic distances (see scale at the bottom of figure).

In addition, the UPGMA tree rooted with *M. cordata* revealed that populations of *M. micrantha* from different locations usually were intermingled (Figure 3), suggesting a lack of geographic pattern. HK87, which was documented as the earliest introduced population in China [49], formed an independent branch, one that first diverged from the other *M. micrantha* populations.

### Detection of signatures of positive selection

**Figure 4:**
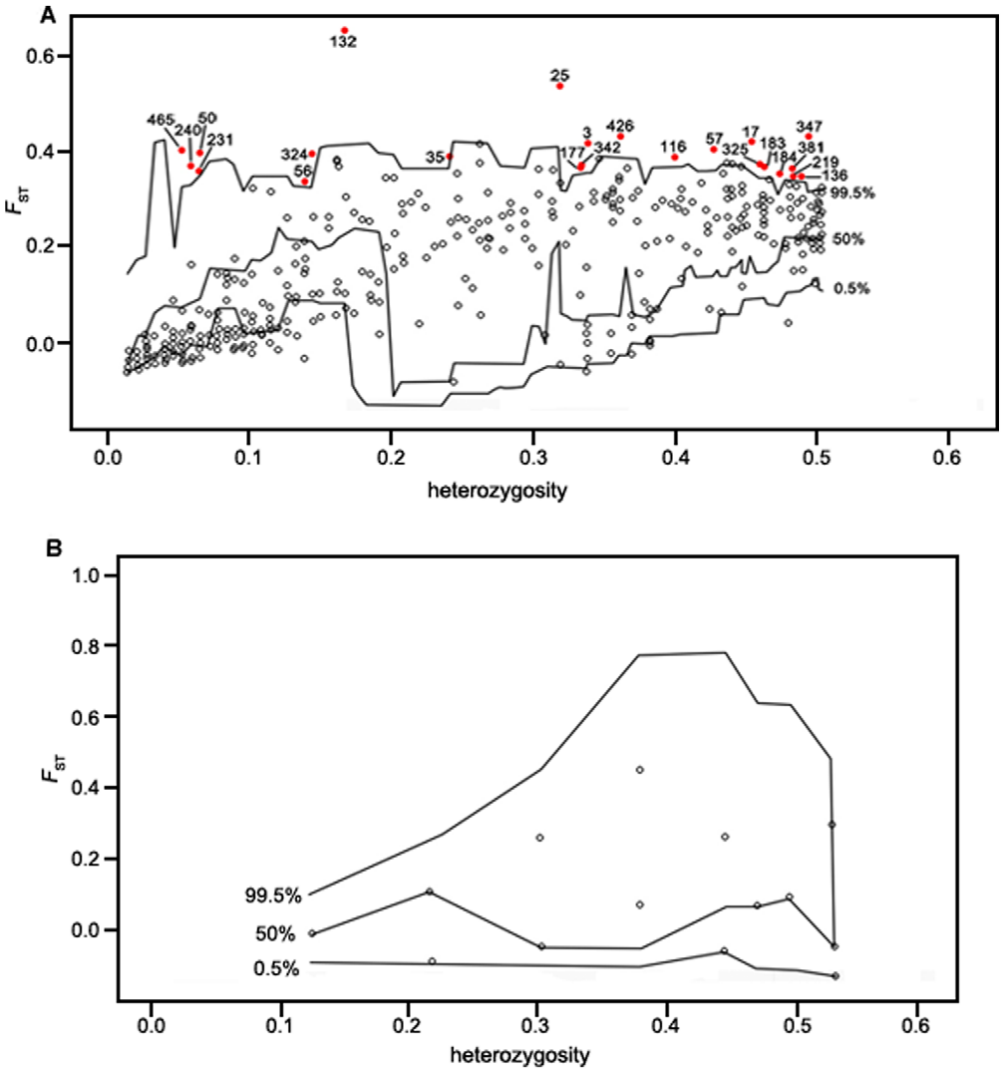
Results of the simulations with Dfdist for outlier detection. Plots representing *F_ST_* values are against heterozygosity. Each dot indicates an AFLP locus. The lower, intermediate, and higher lines represent the 0.5%, 50%, and 99.5% confidence intervals, respectively. Loci above the 99.5% line are regarded as outlier loci. (A) The result of *Mikania micrantha*. The 23 outlier loci under selection are represented by red dots accompanied by the locus number. (B) The result of *Mikania cordata*. No outlier locus under selection is detected.

**Figure 5:**
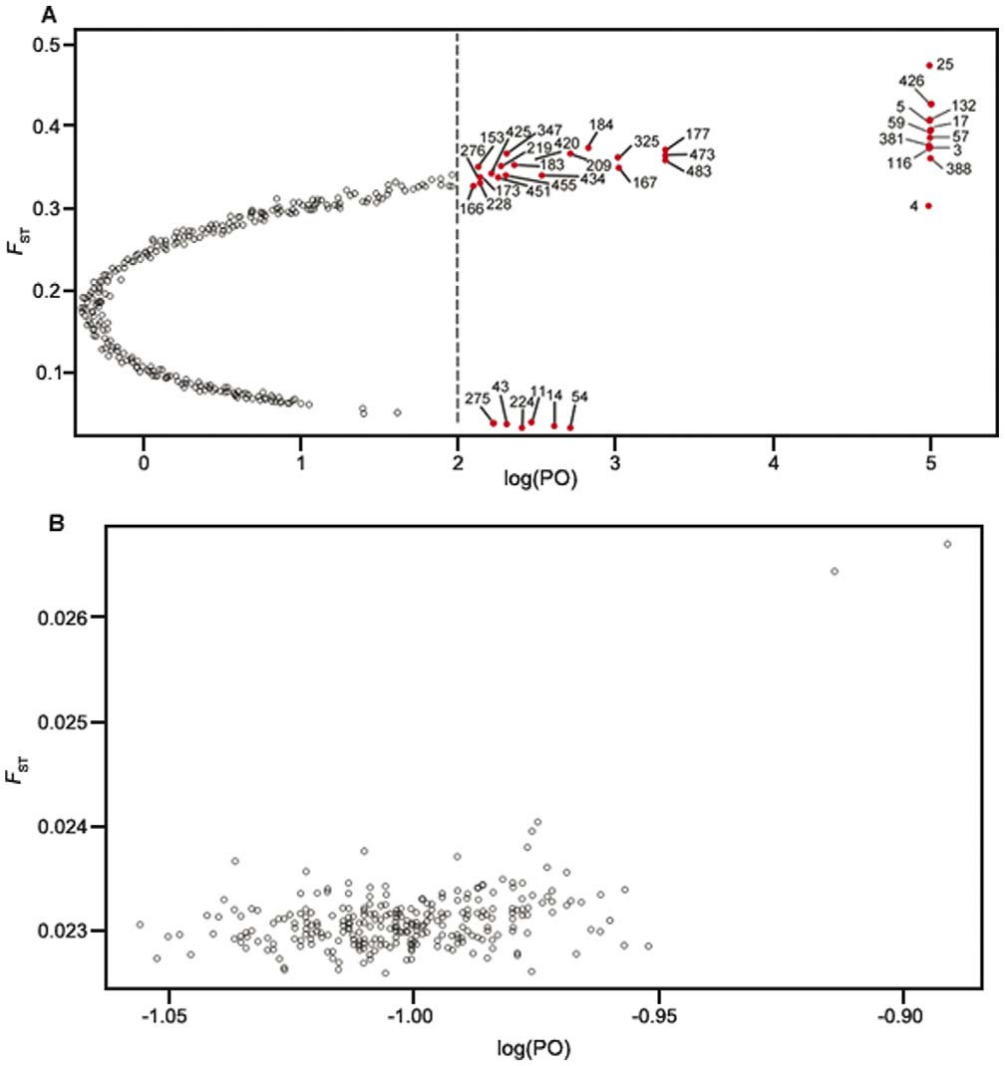
Genomic scan to identify outlier loci subject to selection by Bayescan approach. Each point corresponds to an AFLP locus. *F_ST_* is plotted against the log_10_ of the posterior odds (PO), which provides evidence whether the locus is subject to selection or not. The vertical dashed line shows the decisive threshold value (log_10_ PO = 2.0) used for identifying outlier loci. (A) The result of *Mikania micrantha*. Shown as red dots, the 38 outlier loci with the corresponding locus number are candidates for being under positive selection. (B) The result of *Mikania cordata*. No outlier locus under selection is detected.

The AFLP data set of *M. micrantha* was used in a global analysis for outlier detection, both with Dfdist and Bayescan (Table 5). By using Dfdist on the 28 populations of *M. micrantha*, 23 out of 483 loci (4.8%) were identified as outlier loci under directional selection at the 99.5% probability level (Figure 4A). Bayescan analysis produced high differentiation loci at a threshold of log_10_ PO>2.0 (posterior probabilities higher than 0.99), corresponding to 7.87% of the 483 investigated loci. This approach identified 38 outlier loci potentially under selection or linked to a locus under selection (Table 5; Figure 5A). Notably, Bayescan found 24 outliers (loci 4, 5, 11, 14, 43, 54, 59, 153, 166, 167, 173, 209, 224, 228, 275, 276, 388, 420, 425, 434, 451, 455, 473, and 483) that were not detected by Dfdist. By contrast, Dfdist detected nine outliers (loci 35, 50, 56, 136, 231, 240, 324, 342, and 465) not identified by Bayescan. By pooling the results of the totally different two detection approaches, 14 outlier loci (3, 17, 25, 57, 116, 132, 177, 183, 184, 219, 325, 347, 381 and 426) were identified by Dfdist and Bayescan. The 14 outliers represented truly adaptive loci (not false positives) because the two approaches used different algorithms and assumptions, and very stringent significance criteria were considered (99.5% probability level for Dfdist, posterior probability >0.90 for Bayescan) in the study. Additionally, in the two populations of *M. cordata*, no outlier locus under selection was detected by either Dfdist or Bayescan (Figures 4B and 5B). Nevertheless, this lack of detection of any outliers may also be due to lower sample size.

**Table 5:** Comparison of outlier loci of *Mikania micrantha* under selection using Bayescan, Dfdist, and both with Dfdist and Bayescan, respectively.

Approach	Outiler loci identified
Bayescan	3, 4, 5, 11, 14, 17, 25, 43, 54, 57, 59, 116, 132, 153, 166, 167, 173, 177, 183, 184, 209, 219, 224, 228, 275, 276, 325, 347, 381, 388, 420, 425, 426, 434, 451, 455, 473, 483
Dfdist	3, 17, 25, 35, 50, 56, 57, 116, 132, 136, 177, 183, 184, 219, 231, 240, 324, 325, 342, 347, 381, 426, 465
Bayescan and Dfdist	3, 17, 25, 57, 116, 132, 177, 183, 184, 219, 325, 347, 381, 426

## Discussion

To the best of our knowledge, this study is the first report on detection of candidate loci under selection by genome scan in the invasive weed *M. micrantha* since its introduction to southern China. To avoid spurious outlier loci, several authors advocated employment of two or more outlier detection methods and a conservative significance level [25], [28], [62]–[65]. In this study, the AFLP explorative genome scan has revealed 14 loci as under selection among a total of 483 loci in *M. micrantha*. These 14 loci are considered to possess high credibility because they were picked up by two complementary and exhaustive methods, Dfdist and Bayescan, applying very stringent significance criteria and a 30% trimmed mean *F*_ST_. Meyer *et al.* noted that the power of the analysis is directly associated with the genome coverage [23]. Since a high density of AFLP loci were examined, the selective loci detected here should prove to have good reliability. Taking these together, the 14 outlier loci we identified can be considered useful for understanding the successful invasion of *M. micrantha* in southern China.

Currently, there is increasing interest in identifying genes or outlier loci that underlie local adaptations in invasive species [66]–[68]. Understanding the process of invasive adaptive evolution is critical to the introduction history and genetic consequences on introduced populations, the planning of management strategies of invasive species, and for identifying the possible risks of introduced non-native species in the future [2], [30]. Because selection for local adaptation is unlikely to simultaneously occur at many loci [69], outlier loci are limited to a minor part of the genome, which has been suggested in other species [28], [63], [69]–[71]. Our results indicate that only a small part (2.9%) of the genome of *M. micrantha* has been under directional selection during its invasive processes. The percentage of loci detected is slightly lower than the 5–10% reported in the generality of AFLP genome scans [72], [73]. As AFLP loci are likely located in non-coding DNA, some of the outlier loci may only exhibit the signature of selection because they are linked to the actual target [26], [37]. Although it is difficult to know the location and function of the loci involved in the adaptation to invasiveness, a genome scan of *M. micrantha* still offers unique opportunity to unravel the genetic basis of invasive adaptation without known phenotypes and whole genome sequences. In particular, the AFLP primers that were developed to amplify the outlier loci identified here can be directly used to construct a reduced representation library of the *M. micrantha* genome, which will allow efficient sequencing of the linked genomic regions by next-generation sequencing technology [74].

Genetic diversity is anticipated to increase the adaptive potential of invasive populations in the new environment [4], [9]. In invasive species, the alleles from standing genetic variation have been supposed to control the adaptive evolution [29], [30], because favorable alleles compared to neutral or deleterious alleles are immediately available and often occur at a greater frequency in populations [19], [30]. Compared with other non-invasive and invasive weedy species (Table 6), we have detected relatively high levels of genetic diversity in the introduced populations of *M. micrantha* in southern China. This substantial genetic variation enables to provide a large pool of raw material for adaptive evolution [30], [67].

**Table 6:** Percentage of polymorphic loci (*PL*), Nei's total gene diversity (*H*_T_), *G_ST_*, *Φ_ST_*, and *θ^B^* obtained from the populations of different weedy species based on AFLP data.

Species	*PL*	*H*_T_	*G_ST_*	*Φ_ST_*	*θ^B^*	Reference
*Guizotia scabra* ssp. *scabrai*	0.8455	0.32	0.18	0.22		Geleta et al. [130]
*Guizotia scabra* ssp. *schimperi*	0.9002	0.32	0.19	0.17		Geleta et al. [130]
*Guizotia villosa*	0.8393	0.33	0.19	0.26		Geleta et al. [130]
*Nicotiana attenuata*	0.961			0.114	0.0549	Bahulikar et al. [131]
*Scalesia affinis*	0.549				0.435	Nielsen [132]
*Oxalis pes-caprae* *	0.8843			0.23		Rottenberg & Parker [94]
*Festuca pratensis*	0.9320			0.31		Fjellheim & Rognli [133]
*Senecio vulgaris*	0.34			0.1808		Haldimann et al. [134]
*Ranunculus glacialis*	0.99					Schönswetter et al. [135]
*Ranunculus carpaticola*	0.8594			0.7442		Paun et al. [136]
*Arabidopis thaliana*	1.00	0.179	0.279	0.23		Jørgensen & Mauricio [137]
*Lolium perenne*	0.886					Treuren et al. [138]
*Alopecurus myosuroides*	0.905					Menchari et al. [139]
*Mikania micrantha*	1.00	0.3376	0.4736	0.3335	0.2927	This Study

* Invasive weed.

High genetic diversity can be created by multiple introductions, which bring together large amounts of genetic variation and novel genetic combinations [8]. Such admixtures elevate the expansive ability and pace of proliferation of invasive species and are able to enhance adaptive evolutionary responses to novel environmental selection [10], [12], [19], [75]. Based on the UPGMA and STRUCTURE analysis results, closely related populations often are from different geographical locations (Figures 2 and 3), while individuals from the same location do not group together. Thus multiple introductions are inferred among populations of *M. micrantha*. In addition, the significant genetic differentiation detected among *M. micrantha* populations is also consistent with the expectation that each population has experienced severe bottleneck (Table 3). It is generally suggested that bottlenecks tend to increase genetic differentiation among populations by changing allelic frequencies [76], [77] and they are perceived to reduce the potential for adaptive evolution as well [29], [78]. Nevertheless, multiple introductions can ameliorate this effect and accelerate the speed of adaptive evolution [8]. Such phenomena have been recorded in the populations of other invasive species including *Heracleum mantegazzianum* [79], *Hieracium lepidulum* [80], and *Alliaria petiolata* [81]. Furthermore, no geographical signature is revealed in the AFLP variation pattern among the introduced populations of *M. micrantha* at either regional or entire range scale. Repeated or secondary introductions, which are induced by long-distance dispersals of copious amounts of fine and fluffy seeds, pollination or propagules mediated by humans, insects, and violent winds [82], may further complicate the geographic consequences of the population genetic variation of *M. micrantha*. Therefore, multiple introductions can add to the successful invasiveness of *M. micrantha* by increasing genetic variation, preventing genetic bottlenecks, and strengthening the capacity of adaptive evolution.

Adaptive evolution of invasive species is also determined by the reproductive system such as the amount of sexual and asexual reproduction and the patterns of mating [19]. *Mikania micrantha* mainly propagates by seeds, but its local spread mostly results from vegetative propagation [56], [83], [84]. When reproducing vegetatively, *M. micrantha* generates shoots from small stem fragments and rosettes [83], [84]. In this study, significant LD and locus compatibility have been identified in a majority of *M. micrantha* populations (Table 2), indicating that asexual reproduction is the predominant propagation system. In addition, self-fertilization (selfing) has also been reported in the southern China populations of *M. micrantha* [85]. The results are in accord with the findings that most invasive plants reproduce asexually or by selfing [86]–[88], and reproductive systems may be subject to evolutionary modification during invasion such as switching to higher levels of inbreeding or vegetative reproduction [5], [19], [88]–[91]. For species that are able to reproduce both sexually and clonally, sexual reproduction would promote allele recombinations that result in high genetic variation, whereas clonal reproduction preserves successful combinations and maintains genetic variation by occasionally creating new alleles through somatic mutations [92]–[95]. The specific reproductive modes (asexual and selfing) of *M. micrantha* are likely to enhance its adaptive potential in southern China.

In summary, this study is the first report to identify outlier loci under selection in the genome of the invasive weed *M. micrantha* without reference to coding vs. noncoding sequences, or specific models of selection by explorative genomic scans [25]. We find that 2.9% candidate loci are under selection, indicating that local adaptation has indeed occurred in *M. micrantha* since its introduction to southern China. This study also demonstrates high genetic variability, strong genetic differentiation, severe bottlenecks, and multiple introductions, as well as primarily asexual reproduction in the introduced populations. The interactions between genetic diversity, multiple introductions, and reproductive modes are revealed to be involved in the response of *M. micrantha* to the new local environment, allowing a more precise understanding of its successful invasion. In future studies, we will isolate and sequence the outlier AFLP bands to identify their genomic locations and neighboring genes to further detail the adaptive molecular mechanisms of *M. micrantha*.

## Materials and Methods

### Ethics statement

This study was conducted in accordance with all People's Republic of China laws. No specific permits were required for the described field studies. No specific permissions were required for the locations/activities described in this study. The location is not privately owned or protected in any way. The field studies did not involve endangered or protected species.

### Plant material

For *M. micrantha*, individuals were sampled from 28 populations in six regions (Hong Kong, Macao, Shenzhen, Neilingding, Zhuhai, and Dongguan) representing different habitats (Figure 1A; Table 1). In each population, we collected fresh leaf material from 12 to 19 randomly selected individuals (Table 1). These samples represented a large number of individuals of *M. micrantha* throughout its introduced range in southern China. To further confirm that the outlier loci under selection in *M. micrantha* were caused by novel environments, we tested whether AFLP loci can demonstrate evidence of selection in the congeneric species *M. cordata*, which is native to sub-tropical China. For *M. cordata*, five to 12 individuals were collected from its two populations (HN1 and HN2) located on Hainan Island (Figure 1B; Table 1). Leaves were preserved in silica gel. Voucher specimens (*M. micrantha*: CGP1-409; *M. cordata*: CGP410-426) were deposited at the herbarium of Sun Yat-sen University (SYS), Guangzhou, China.

### DNA extraction

Total genomic DNA was extracted from ground tissue, following the modified CTAB protocol [96]. The quality and quantity of the DNA were determined with a spectrophotometer (Pharmacia 2000 UV/Visible, Amersham Pharmacia Biotech, Piscataway, NJ) and on 0.8% agarose gels.

### AFLP protocols

AFLP analyses were performed according to Vos *et al.* [97] with the following modifications: genomic DNA (50 ng) was digested with 2 U EcoRI and 4 U MseI (New England Biolabs, Ipswitch, MA) for 3 h at 37°C in 1×NE buffer and incubated at 70°C for 20 min. To ligate the resulting fragments to the corresponding adapters, 10 µl of restriction products were added into a 20 µl reaction mixture containing 0.1 µM EcoRI adapter, 1 µM MseI adapter, and 60 U T4 DNA ligase (New England Biolabs). After being incubated at 20°C for 3 h, the samples were diluted 10 times with ddH_2_O, then 2 µl of each sample was used as a template to conduct the pre-amplification in a final volume of 20 µl that contained 1×PCR buffer, 100 nM each of EcoRI+A and MseI+C primers, 0.24 mM dNTPs, and 1.5 U *Taq* DNA polymerase. The preamplification reaction was carried out for 20 cycles of 30 s at 94°C, 1 min at 56°C and 1 min at 72°C. After that, preamplified product was diluted 1∶50 with ddH_2_O, and then used as template for the selective PCR amplifications to generate AFLPs.

Selective amplification was performed in a final volume of 10 µl containing 1× PCR buffer, 125 nM EcoRI primer, 125 nM 6-FAM-EcoRI primer, 250 nM MseI primer, 0.2 mM dNTPs, 0.75 U *Taq* DNA polymerase and 2 µl diluted pre-amplified DNA sample. The reaction was conducted for 13 cycles of 30 s at 94°C, 30 s at 65°C and 1 min at 72°C. The annealing temperature was reduced by 0.7°C per cycle. Then 23 cycles consisting of 30 s at 94°C, 30 s at 56°C, and 1 min at 72°C were performed.

An initial screening of 48 combinations of selective primers, in which six were EcoRI (EcoRI+AAG, +ACT, +AGC, +ACA, +AAC, +ACC) and eight were MseI (MseI+CAG, +CTC, +CTA, +CAA, +CAC, +CTG, +CAT, +CTT), were performed. Eight combinations were selected that generated clear and evenly distributed bands: EcoRI+ACT/MseI+CAG, EcoRI+AAC/MseI+CAG, EcoRI+ACT/MseI+CTA, EcoRI+AAC/MseI+CTC, EcoRI+AAC/MseI+CTA, EcoRI+ACT/MseI+CTC, EcoRI+AGC/MseI+CAC, and EcoRI+ACC/MseI+CAC. Therefore, the eight primer combinations were chosen for selective amplifications of all samples. PCR reactions were conducted on a PTC-100 Peltier Thermal Cycler (MJ Research, St. Bruno, Quebec). The selective PCR products were separated by electrophoresis on 6.5% polyacrylamide gels on an ABI 377 automated sequencer (Applied Biosystems, Carlsbad, CA). An ROX-500-labeled internal size standard (Applied Biosystems) was added to each sample to size fragments.

### Data analysis

Software GeneScan 3.7 (Applied Biosystems) and genographer (version 1.6.0; http://hordeum.oscs.montana.edu/genographer) were utilized to collect and score raw fluorescent AFLP data. The presence (1) or absence (0) of data from unambiguous AFLP bands was used to establish the matrix of genetic identity of the sampled individuals. Genetic diversity statistics, including percentage of polymorphic loci and Nei's gene diversity [98], were calculated using POPGEN32 software [99]. Shannon's index of phenotypic diversity was quantified as S = −∑ pilnpi where pi is the frequency of a given AFLP band in the population [100].

Analysis of Molecular Variance (AMOVA) based on a Euclidean squared distance matrix was hierarchically calculated to estimate the allocation of genetic variation among and within populations by ARLEQUIN 3.0 (available at http://cmpg.unibe.ch/software/arlequin3) [101]. In this analysis, the AFLP data set was partitioned at three levels: regional, among- population, and within- population. One thousand random permutations were used to infer the significance of the variance components [101]. Also using the same software, a Mantel test was performed to investigate the correlation between genetic differentiation and geographic distances (km) among populations. The matrix of genetic differentiation was composed by pairwise *θ^B^* estimates.

Holsinger *et al.* [102] proposed a Bayesian approach to estimating genetic structure for dominant and co-dominant markers. The nearly unbiased parameter estimations of heterozygosity, genetic distance, and population differentiation can be obtained using the Bayesian method [102]. Its *f* and *θ^B^* are equivalent to the inbreeding coefficient (*F*_IS_) and the fixation index (*F*_ST_) of F-statistics, respectively. The posterior distributions of *f* and *θ^B^* were estimated through Markov Chain Monte Carlo (MCMC) methods by HICKORY v1.0, with a burn-in of 50 000 iterations and a sampling run of 250 000 iterations from which every fiftieth sample was retained for posterior calculations [102]. The analysis model was chosen based on the deviance information criterion (DIC). The “*f*-free” model, where *f* was not estimated but was chosen at random from the prior distribution, was decided due to its smaller DIC than for other models. Alleles were deemed rare if their frequencies were ≤0.05 in the sampled populations [59].

To gain further perspectives on genetic structure, we also employed the Bayesian clustering method to infer the pattern of population structure by implementing the STRUCTURE 2.2.3 program [103], [104]. To determine the best number of clusters, 30 independent runs of *K* (*K* = 1 to 30) were performed with an admixture model at 100 000 MCMC iterations and a 10 000 burn-in period. We used *ΔK*, the second-order rate of change in ln P (*X*|*K*) for successive values of *K* to determine the number of clusters [61]. The distribution map of STRUCTURE was plotted according to *K* value at the highest log likelihood. Moreover, based on Nei's genetic distance [105], an unweighted pair group method with arithmetic mean analysis (UPGMA) was used to generate a dendrogram of the relationships among the populations by TFPGA 1.3 [106]. One thousand bootstrap replicates were permuted to assess the reliability of the UPGMA dendrogram.

We further tested whether populations have suffered a bottleneck using the program BOTTLENECK [107]. The heterozygosity (Heq) expected at mutation-drift equilibrium was calculated based on both the stepwise mutation model (SMM) and the infinite allele model (IAM) [108], [109]. The significance of heterozygosity excess was determined by the sign test [110].

Linkage disequilibrium, which is an association of alleles at different loci on chromosomes in a population, is mainly affect by population size [111]. The dependency of population size can be removed by multilocus linkage disequilibrium with both indices of association *I*_A_ [112]–[114] and its modified measure 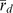 [115]. These indices and their significance by randomization were computed using the software Multilocus v1.2 (http://www.bio.ic.ac.uk/evolve/software/multilocus/).

A character incompatibility analysis was carried out to probe the predominant mating system in the populations at the molecular level [116]–[118]. In a pair of binary character data, such as the presence or absence of AFLP bands at two loci, the presence of all four possible combinations of characters (0/0, 1/0, 0/1, 1/1) is more parsimoniously explained by sexual recombination than by three mutation events. Once all four character combinations arise, an incompatibility is said to occur, and can be used as a measure of recombination [116]. The incompatibility excess ratio (IER) was calculated using PICA 4.0 by comparing the observed incompatibility count for the original data and mean incompatibility count for randomly permuted data [118]. If none or a small fraction of incompatible loci occur, asexual reproduction is implied to have happened [80], [119].

To detect outlier loci under selection for local adaptation of *M. micrantha* and *M. cordata*, two complementary methods were applied to our AFLP data set. We first used Program Dfdist, which is the most popular software for detecting candidate loci [120]. Dfdist software (http://www.rubic.rdg.ac.uk/~mab/stuff) was recently modified from Beaumont and Balding [43] to analyze dominant data, which is a hierarchical Bayesian approach based on summary statistics in a symmetrical island model [24], [39]. Outlier loci were detected by comparing empirical *F*_ST_ values for each locus against a null distribution of *F*_ST_ values expected from a neutral drift model [34]. A potential defect of the Dfdist approach for detecting outlier loci is the possibility of false positives [121]–[123]. The statistical power was enhanced to avoid false positives by setting a conservative constraint [22]. A null distribution of *F*_ST_ close to the empirical distribution was acquired by 50 000 coalescent simulations. Simulations were computed with a mean *F*_ST_ similar to the trimmed mean *F*_ST_, which was calculated by excluding 30% of the most extreme *F*_ST_ values observed in the empirical dataset [71], [124]. The 4Nμ parameter value was set to 0.04 in all simulations. A global analysis was done for *M. micrantha* and *M. micrantha* using 28 populations and 2 populations, respectively. The threshold for outliers was set to the more conservative 0.005, estimated from simulated *F*_ST_ values to control for false positives [28], [33], [125]. In addition, Dfdist assumes that populations are at migration-drift equilibrium, which does not often occur in natural populations [124]. Hence, to cross-check the reliability of the outlier loci detected by Dfdist, we also ran Bayescan software (http://www-leca.ujf-grenoble.fr/logiciels.htm), which better handles dominant marker data by directly estimating the posterior probability of a given locus to be under selection [42]. Assuming that allele frequencies within populations follow a Dirichlet distribution [126]–[128], the Bayesian method not only permits for different demographic scenarios and different amounts of genetic drift between populations when estimating population-specific *F*_ST_ coefficients, but also considers all loci in the analyses [124]. The Bayesian approach also disposes of the problem of multiple testing of a large number of genomic loci through prior distribution [124]. In our genome scan, the log_10_ PO>2.0 was considered a threshold value for determining loci under selection according to Jeffreys' interpretation [129], which is a logarithmic scale for model choice as follows: log_10_ PO>0.5 (substantial); log_10_ PO>1.0 (strong); log_10_ PO>1.5 (very strong); and log_10_ PO>2.0 (decisive support for accepting a model) [36]. We used 10 pilot runs of 5000 iterations to estimate model parameters. A burn-in of 50 000 iterations was employed to cover the MCMC. The sample size was set to 5000 and the thinning interval to 20, resulting in a total chain length of 150 000 iterations [45]. The loci were ranked according to their estimated posterior probability and all loci with a value over 0.993 were retained as outliers. This corresponds to log_10_ PO>2.0, which provides decisive support for acceptation of the model. Outliers identified by both Dfdist and Bayescan are likely to be truly adaptive regions of the genome, because the two approaches differ in algorithms and assumptions [27].

## References

[1] Holt RD (2009). Up against the edge: invasive species as testbeds for basic questions about evolution in heterogeneous environments.. Mol Ecol.

[2] Xu CY, Julien MH, Fatemi M, Girod C, Van Klinken RD (2010). Phenotypic divergence during the invasion of *Phyla canescens* in Australia and France: evidence for selection-driven evolution.. Ecol Lett.

[3] Richardson DM, Pyšek P, Rejmánek M, Barbour MG, Panetta FD (2000). Naturalization and invasion of alien plants: concepts and definitions.. Divers Distrib.

[4] Lachmuth S, Durka W, Schurr FM (2010). The making of a rapid plant invader: genetic diversity and differentiation in the native and invaded range of *Senecio inaequidens*.. Mol Ecol.

[5] Barrett SCH, Husband B, Brown AHD, Clegg MT, Kahler AL, Weir BS (1990). The genetics of plant migration and colonization.. Plant population genetics, breeding, and genetic resources.

[6] Novak SJ, Mack RN (1993). Genetic variation in *Bromus tectorum* (Poaceae): comparison between native and introduced populations.. Heredity.

[7] Milne RI, Abbott RJ (2000). Origin and evolution of invasive naturalized material of *Rhododendron ponticum* L. in the British Isles.. Mol Ecol.

[8] Dlugosch MK, Parker MI (2008). Founding events in species invasions: genetic variation, adaptive evolution, and the role of multiple introductions.. Mol Ecol.

[9] Sakai AK, Allendorf FW, Holt JS, Lodge DM, Molofsky J (2001). The population biology of invasive species.. Annu Rev Ecol Sys.

[10] Lee CE (2002). Evolutionary genetics of invasive species.. Trends Ecol Evol.

[11] Holt RD, Barfield M, Gomulkiewicz R, Sax DF, Stachowicz JJ, Gaines SD (2005). Theories of niche conservatism and evolution: could exotic species be potential tests?. Species invasions: insights into ecology, evolution, and biogeography.

[12] Keller SR, Taylor DR (2008). History, chance and adaptation during biological invasion: separating stochastic phenotypic evolution from response to selection.. Ecol Lett.

[13] Allan E, Pannell JR (2009). Rapid divergence in physiological and life-history traits between northern and southern populations of the British introduced neo-species, *Senecio squalidus*.. Oikos.

[14] Reznick DN, Ghalambor CK (2001). The population ecology of contemporary adaptations: what empirical studies reveal about the conditions that promote adaptive evolution.. Genetica.

[15] Whitney KD, Gabler CA (2008). Rapid evolution in introduced species, ‘invasive traits’ and recipient communities: challenges for predicting invasive potential.. Divers Distrib.

[16] Kaufman SR, Smouse PE (2001). Comparing indigenous and introduced populations of *Melaleuca quinquenervia* (Cav.) Blake: response of seedlings to water and pH levels.. Oecologia.

[17] Broennimann O, Treier UA, Müller-Scharer H, Thuiller W, Peterson AT (2007). Evidence of climatic niche shift during biological invasion.. Ecol Lett.

[18] Kanarek AR, Webb CT (2010). Allee effects, adaptive evolution, and invasion success.. Evol Appl.

[19] Barrett SCH, Colautti RI, Eckert CG (2008). Plant reproductive systems and evolution during biological invasion.. Mol Ecol.

[20] Wang JY, Zhang HW, Huang RF (2007). Expression analysis of low temperature responsive genes in *Eupatorium adenophorum* Spreng using cDNA-AFLP.. Plant Mol Biol Rep.

[21] Pyŝek P (1998). Is there a taxonomic pattern to plant invasions?. Oikos.

[22] Herrera CM, Bazaga P (2009). Quantifying the genetic component of phenotypic variation in unpedigreed wild plants: tailoring genomic scan for within-population use.. Mol Ecol.

[23] Meyer CL, Vitalis R, Saumitou-Laprade P, Castric V (2009). Genomic pattern of adaptive divergence in *Arabidopsis halleri*, a model species for tolerance to heavy metal.. Mol Ecol.

[24] Bonin A, Taberlet P, Miaud C, Pompanon F (2006). Explorative genome scan to detect candidate loci for adaptation along a gradient of altitude in the common frog (*Rana temporaria*).. Mol Biol Evol.

[25] Murray MC, Hare MP (2006). A genomic scan for divergent selection in a secondary contact zone between Atlantic and Gulf of Mexico oysters, *Crassostrea virginica*.. Mol Ecol.

[26] Schlötterer C (2003). Hitchhiking mapping-functional genomics from the population genetics perspective.. Trends Genet.

[27] Paris M, Boyer S, Bonin A, Collado A, David JP (2010). Genome scan in the mosquito *Aedes rusticus*: population structure and detection of positive selection after insecticide treatment.. Mole Ecol.

[28] Galindo J, Morán P, Rolán-Alvaren E (2009). Comparing geographical genetic differentiation between candidate and noncandidate loci for adaptation strengthens support for parallel ecological divergence in the marine snail *Littorina saxatilis*.. Mol Ecol.

[29] Prentis PJ, Wilson JRU, Dormontt EE, Richardson DM, Lowe AJ (2008). Adaptive evolution in invasive species.. Trends Plant Sci.

[30] Prentis PJ, Woolfit M, Thomas-Hall SR, Ortiz-Barrientos, Pavasovic A (2010). Massively parallel sequencing and analysis of expressed sequence tags in a successful invasive plant.. Ann Bot.

[31] Mealor BA, Hild AL (2006). Potential selection in native grass populations by exotic invasion.. Mol Ecol.

[32] Campbell D, Bernatchez L (2004). Generic scan using AFLP markers as a means to assess the role of directional selection in the divergence of sympatric whitefish ecotypes.. Mol Biol Evol.

[33] Gagnaire PA, Albert V, Jónsson B, Bernatchez L (2009). Natural selection influences AFLP intraspecific genetic variability and introgression patterns in Atlantic eels.. Mol Ecol.

[34] Michalski SG, Durka W, Jentsch A, Kreyling J, Pompe S (2010). Evidence for genetic differentiation and divergent selection in an autotetraploid forage grass (*Arrhenatherum elatius*).. Theor Appl Genet.

[35] Parisod C, Joost S (2010). Divergent selection in trailing-versus leading-edge populations of *Biscutella laevigata*.. Ann Bot.

[36] Fischer MC, Foll M, Excoffier L, Heckel G (2011). Enhanced AFLP genome scans detect local adaptation in high-altitude populations of a small rodent (*Microtus arvalis*).. Mol Ecol.

[37] Tollenaere C, Duplantier JM, Rahalison L, Ranjalahy M, Brouat C (2011). AFLP genome scan in the black rat (*Rattus rattus*) from Madagascar: detecting genetic markers undergoing plague-mediated selection.. Mol Ecol.

[38] Beaumont MA, Nichols RA (1996). Evaluating loci for use in the genetic analysis of population structure.. Proc Roy Soc B-Biol Sci.

[39] Wright S (1951). The genetical structure of populations.. Ann Eugen.

[40] Excoffier L, Hofer T, Foll M (2009). Detecting loci under selection in a hierarchically structured population.. Heredity.

[41] Helyar SJ, Hemmer-Hansen J, Bekkevold D, Taylor MI, Ogden R (2011). Application of SNPs for population genetics of nonmodel organisms: new opportunities and challenges.. Mol Ecol Resour.

[42] Foll M, Gaggiotti O (2008). A genome-scan method to identify selected loci appropriate for both dominant and codominant markers: a Bayesian perspective.. Genetics.

[43] Beaumont MA, Balding DJ (2004). Identifying adaptive genetic divergence among populations from genome scans.. Mol Ecol.

[44] Butlin RK (2010). Population genomics and speciation.. Genetica.

[45] Pérez-Figueroa A, García-Pereira MJ, Saura M, Rolán-Alvarez E, Caballero A (2010). Comparing three different methods to detect selective loci using dominant markers.. J Evol Biol.

[46] Holm LG, Plucknett DL, Pancho JV, Herberger JP (1977). The world's worst weeds: distribution and biology.

[47] Wirjahar S (1976). Autecological study of *Mikania* spp.. Proceedings of fifth Asian-Pacific weed science society conference, 5–11, October, 1975.

[48] Kong GH, Wu QG, Hu QM (2000). Exotic weed *Mikania micrantha* H. B. K. appeared in south China.. J Trop Subtrop Bot.

[49] Wang BS, Liao WB, Zan QJ, Li MG, Zhou XY (2003). The spreads of *Mikania micrantha* in China.. Acta Scient Nat Univ Sunyatseni.

[50] Ellison CA, Evans HC, Djeddour DH, Thomas SE (2008). Biology and host range of the rust fungus *Puccinia spegazzinii*: A new classical biological control agent for the invasive, alien weed *Mikania micrantha* in Asia.. Biol Control.

[51] Cock MJW, Ellison CA, Evans HC, Ooi PAC, Spencer NR (2000). Can failure be turned into success for biological control of mile-a-minute weed (*Mikania micrantha*)?. Proceedings of the X international symposium on biological control of weeds.

[52] Huang ZL, Cao HL, Liang XD, Ye WH, Feng HL (2000). The growth and damaging effect of *Mikania micrantha* in different habitats.. J Trop Subtrop Bot.

[53] Ismail BS, Chong TV (2002). Effects of aqueous extracts and decomposition of *Mikania micrantha* H. B. K. debris on selected agronomic crops.. Weed Biol Manag.

[54] Ni GY, Song LY, Zhang JL, Peng SL (2006). Effects of root extracts of *Mikania micrantha* H. B. K. on soil microbial community.. Allelopathy J.

[55] Zan QJ, Wang YJ, Wang BS, Liao WB, Li MG (2000). The distribution and harm of the exotic weed *Mikania micrantha*.. Chinese J Ecol.

[56] Zhang LY, Ye WH, Cao HL, Feng HL (2004). *Mikania micrantha* H. B. K. in China – an overview.. Weed Res.

[57] Maron JL, Vilà M, Bommarco R, Elmendorf S, Beardsley P (2004). Rapid evolution of an invasive plant.. Ecol Monogr.

[58] Baker SA, Dyer RJ (2011). Invasion genetics of *Microstegium vimineum* (Poaceae) within the James River Basin of Virginia, USA.. Conserv Genet.

[59] Marshall DR, Brown ADH, Frankel OH, Hawkes JG (1975). Optimum sampling strategies for gene conservation.. Crop genetic resources for today and tomorrow.

[60] Wright S (1943). Isolation by distance.. Genetics.

[61] Evanno G, Regnaut S, Goudet J (2005). Detecting the number of clusters of individuals using the software STRUCTURE: a simulation study.. Mol Ecol.

[62] Vasemägi A, Primmer CR (2005). Challenges for identifying functionally important genetic variation: the promise of combining complementary research strategies.. Mol Ecol.

[63] Egan SP, Nosil P, Funk DJ (2008). Selection and genomic differentiation during ecological speciation: isolating the contributions of host association via a comparative genome scan of *Neochlamisus bebbianae* leaf beetles.. Evolution.

[64] Williams MW, Oleksiak MF (2008). Signatures of selection in natural populations adapted to chronic pollution.. BMC Evol Biol.

[65] Tice KA, Carlon DB (2011). Can AFLP genome scans detect small islands of differentiation? The case of shell sculpture variation in the periwinkle *Echinolittorina hawaiiensis*.. J Evol Biol.

[66] Pimental D, Lach L, Zuniga R, Morrison D (2000). Environmental and economic costs of nonindigenous species in the United States.. BioScience.

[67] Rieseberg LH, Kim SC, Randell RA, Whitney KD, Gross BL (2007). Hybridization and the colonization of novel habitats by annual sunflowers.. Genetica.

[68] Shimada Y, Shikano T, Merila J (2011). A high incidence of selection on physiologically important genes in the three-spined stickleback, *Gasterosteus aculeatus*.. Mol Biol Evol.

[69] Kuchma O, Finkeldey R (2011). Evidence for selection in response to radiation exposure: *Pinus sylvestris* in the Chernobyl exclusion zone.. Environ Pollut.

[70] Nosil P, Egan SP, Funk DJ (2008). Heterogeneous genomic differentiation between walking stick ecotypes: “Isolation by adaptation” and multiple roles for divergent selection.. Evolution.

[71] Smith TB, Milá B, Grether GF, Slabbekoorn H, Sepil I (2008). Evolutionary consequences of human disturbance in a rainforest bird species from Central Africa.. Mol Ecol.

[72] Nunes VR, Beaumont MA, Butlin RK, Paulo OS (2011). Multiple approaches to detect outliers in a genome scan for selection in ocellated lizards (*Lacerta lepida*) along an environmental gradient.. Mol Ecol.

[73] Nosil P, Funk DJ, Ortiz-Barrientos D (2009). Divergent selection and heterogeneous genomic divergence.. Mol Ecol.

[74] Leroux S, Feve K, Vignoles F, Bouchez O, Klopp C (2010). Non PCR-amplified transcripts and AFLP® fragments as reduced representations of the quail genome for 454 Titanium sequencing.. BMC Res Note.

[75] Novak SJ, Mack RN, Sax DF, Stachowicz JJ, Gaines SD (2005). Genetic bottlenecks in alien plant species: influences of mating systems and introduction dynamics.. Species invasions: insights into ecology, evolution, and biogeography.

[76] Hedrick PW (1999). Perspective: highly variable loci and their interpretation in evolution and conservation.. Evolution.

[77] Hedrick PW (2000). Genetics of Populations.

[78] Van Buskirk J, Willi Y (2006). The change in quantitative genetic variation with inbreeding.. Evolution.

[79] Walker NF, Hulme PE, Hoelzel AR (2003). Population genetics of an invasive species, *Heracleum mantegazzianum*: implications for the role of life history, demographics and independent introductions.. Mol Ecol.

[80] Chapman H, Robson B, Pearson ML (2004). Population genetic structure of a colonising, triploid weed, *Hieracium lepidulum*.. Heredity.

[81] Durka W, Bossdorf O, Prati D, Auge H (2005). Molecular evidence for multiple introductions of garlic mustard (*Alliaria petiolata*, Brassicaceae) to North America.. Mol Ecol.

[82] Zhou XM, Huang BQ (2001). The invasion and control of *Mikania micrantha*.. World Agr.

[83] Swarmy PS, Ramakrishnan PS (1987). Weed potential of *Mikania micrantha* H.B.K. and its control in fallows after shifting agriculture (Jhum) in north-east India.. Agr Ecosyst Environ.

[84] Wen DZ, Ye WH, Feng HL, Cai CX (2000). Comparison of basic photosynthetic characteristics between exotic invader weed *Mikania micrantha* and its companion species.. J Trop Subtrop Bot.

[85] Hong L, Shen H, Ye WH, Cao HL, Wang ZM (2007). Self-incompatibility in *Mikania micrantha* in South China.. Weed Res.

[86] Price SC, Jain SK (1981). Are inbreeders better colonisers?. Oecologia.

[87] Husband BC, Barrett SCH (1991). Colonization history and population genetic structure of *Eichornia paniculata* in Jamaica.. Heredity.

[88] Amsellem L, Noyer JL, Hossaert-Mckey M (2001). Evidence for a switch in the reproductive biology of *Rubus alceifolius* (Rosaceae) towards apomixis, between its native range and its area of introduction.. Am J Bot.

[89] Burdon JJ, Marshall DR (1981). Biological control and the reproductive mode of weeds.. J Appl Ecol.

[90] Barrett SCH, Richardson BJ, Groves RH, Burdon JJ (1986). Genetic attributes of invading species.. Ecology of biological invasions: an Australian perspective.

[91] Pellegrin D, Hauber DP (1999). Isozyme variation among populations of the clonal species, *Phragmites australis* (Cav.) Trin. ex Steudel.. Aquat Bot.

[92] Klekowski EJ, de Kroon H, van Groenendael J (1997). Somatic mutation theory of clonality.. The ecology and evolution of clonal plants.

[93] Houliston GJ, Chapman HM (2004). Reproductive strategy and population variability in the facultative apomict *Hieracium pilosella* (Asteraceae).. Am J Bot.

[94] Rottenberg A, Parker JS (2004). Asexual populations of the invasive weed *Oxalis pes-caprae* are genetically variable.. Proc Roy Soc B-Biol Sci.

[95] Lavergne S, Molofsky J (2007). Increased genetic variation and evolutionary potential drive the success of an invasive grass.. Proc Natl Acad Sci USA.

[96] Su YJ, Wang T, Zheng B, Jiang Y, Chen GP (2005). Genetic differentiation of relictual populations of *Alsophila spinulosa* in southern China inferred from cpDNA *trn*L–F noncoding sequences.. Mol Phylogenet Evol.

[97] Vos P, Hogers R, Bleeker M, Reijans M, van de Lee T (1995). AFLP: a new technique for DNA fingerprinting.. Nuc Acids Res.

[98] Nei M (1973). Analysis of gene diversity in subdivided populations.. Proc Natl Acad Sci USA.

[99] Yeh FC, Yang R (1999). POPGENE version 1.31.. http://www.ualberta.ca/_/fyeh.

[100] Lewontin RC (1973). The apportionment of human diversity.. Evol Biol.

[101] Excoffier L, Laval G, Schneider S (2005). Arlequin ver. 3.0: An integrated software package for population genetics data analysis.. Evolutionary Bioinformatics Online.

[102] Holsinger KE, Lewis PO, Dey DK (2002). A Bayesian approach to inferring population structure from dominant markers.. Mol Ecol.

[103] Pritchard JK, Stephens M, Donnelly P (2000). Inference of population structure using multilocus genotype data.. Genetics.

[104] Falush D, Stephens M, Pritchard JK (2007). Inference of population structure using multilocus genotype data: dominant markers and null alleles.. Mol Ecol Notes.

[105] Nei M (1978). Estimation of average heterozygosity and genetic distance from a small number of individuals.. Genetics.

[106] Miller MP (1997). Tools for Populations Genetic Analyses (TFPGA) 1.3: A Windows program for the analysis of allozyme and molecular population genetic data..

[107] Luikart G, Cornuet JM (1999). BOTTLENECK: a program for detecting recent effective population size reductions from allele data frequencies.. http://www.montpellier.inra.fr/URLB/bottleneck/bottleneck.html.

[108] Di Rienzo A, Peterson AC, Garza JC, Valdes AM, Slatkin M (1994). Mutational processes of simple sequence repeat loci in human populations.. Proc Natl Acad Sci USA.

[109] Godwin ID, Aitken EAB, Smith LW (1997). Application of inter simple sequence repeat (ISSR) markers to plant genetics.. Electrophoresis.

[110] Cornuet JM, Luikart G (1996). Description and power analysis of two tests for detecting recent population bottlenecks from allele frequency data.. Genetics.

[111] Kim YJ, Feng S, Zeng ZB (2008). Measuring and partitioning the high-order linkage disequilibrium by multiple order Markov chains.. Genet Epidemiol.

[112] Brown AHD, Feldman MW, Nevo E (1980). Multilocus structure of natural populations of *Hordeum spontaneum*.. Genetics.

[113] Maynard SJ, Smith NH, O'Rourke M, Spratt BG (1993). How clonal are bacteria?. Proc Natl Acad Sci USA.

[114] Haubold B, Travisano M, Rainey PB, Hudson RR (1998). Detecting linkage disequilibrium in bacterial populations.. Genetics.

[115] Agapow PM, Burt A (2001). Indices of multilocus linkage disequilibrium.. Mol Ecol Notes.

[116] Mes THM (1998). Character compatibility of molecular markers to distinguish asexual and sexual reproduction.. Mol Ecol.

[117] Van Der Hulst RG, Mes TH, Den Nijs JC, Bachmann K (2000). Amplified fragment length polymorphism (AFLP) markers reveal that population structure of triploid dandelions (*Taraxacum officinale*) exhibits both clonality and recombination.. Mol Ecol.

[118] Wilkinson M (2001). PICA 4.0: software and documentation distributed by the Department of Zoology, Natural History Museum, London, UK.. http://www.nhm.ac.uk/research-curation/projects/software/mwphylogeny.html.

[119] Hassel K, Ståstad SM, Gunnarsson U, Söderström L (2005). Genetic variation and structure in the expanding moss *Pogonatum dentatum* (Polytrichaceae) in its area of origin and in a recently colonized area.. Am J Bot.

[120] Caballero A, Quesada H, Rolán-Alvarez E (2008). Impact of amplified fragment length polymorphism size homoplasy on the estimation of population gene diversity and the detection of selective loci.. Genetics.

[121] Luikart G, England PR, Tallmon D, Jordan S, Taberlet P (2003). The power and promise of population genomics: from genotyping to genome typing.. Nat Rev Genet.

[122] Bonin A, Ehrich D, Manel S (2007). Statistical analysis of amplified fragment length polymorphism data: a toolbox for molecular ecologists and evolutionists.. Mol Ecol.

[123] Herrera CM, Bazaga P (2008). Population-genomic approach reveals adaptive floral divergence in discrete populations of a hawk moth pollinated violet.. Mol Ecol.

[124] Manel S, Conord C, Després L (2009). Genome scan to assess the respective role of host-plant and environmental constraints on the adaptation of a widespread insect.. BMC Evol Biol.

[125] Miller N, Ciosi M, Sappington TW, Ratcliffe ST, Spencer JL (2007). Genome scan of *Diabrotica virgifera virgifera* for genetic variation associated with crop rotation tolerance.. J Appl Entomol.

[126] Balding DJ, Nichols RA (1995). A method for quantifying differentiation between populations at multi-allelic loci and its implications for investigating identity and paternity.. Genetica.

[127] Rannala B, Hartigan JA (1996). Estimating gene flow in island populations.. Genet Res.

[128] Balding DJ (2003). Likelihood-based inference for genetic correlation coefficients.. Theor Popul Biol.

[129] Jeffreys H (1961). Theory of probability (third edition).

[130] Geleta M, Bryngelsson T, Bekele E, Dagne K (2007). AFLP and RAPD analyses of genetic diversity of wild and/or weedy *Guizotia* (Asteraceae) from Ethiopia.. Hereditas.

[131] Bahulikar RA, Stanculescu D, Preston CA, Baldwin IT (2004). ISSR and AFLP analysis of the temporal and spatial population structure of the post-fire annual, *Nicotiana attenuata*, in SW Utah.. BMC Ecology.

[132] Nielsen LR (2004). Molecular differentiation within and among island populations of the endemic plant *Scalesia affinis* (Asteraceae) from the Galápagos Islands.. Heredity.

[133] Fjellheim S, Rognli OA (2005). Molecular diversity of local Norwegian meadow fescue (*Festuca pratensis* Huds.) populations and Nordic cultivars-consequences for management and utilisation.. Theor Appl Genet.

[134] Haldimann P, Steinger T, Müller-Schärer H (2003). Low genetic differentiation among seasonal cohorts in *Senecio vulgaris* as revealed by amplified fragment length polymorphism analysis.. Mol Ecol.

[135] Schöswetter P, Paun O, Tribsch A, Niklfeld H (2003). Out of the Alps: colonization of Northern Europe by East Alpine populations of the Glacier Buttercup *Ranunculus glacialis* L. (Ranunculaceae).. Mol Ecol.

[136] Paun O, Greilhuber J, Temsch EM, Hörandl E (2006). Patterns, sources and ecological implications of clonal diversity in apomictic *Ranunculus carpaticola* (*Ranunculus auricomus* complex, Ranunculaceae).. Mol Ecol.

[137] Jørgensen S, Mauricio R (2004). Neutral genetic variation among wild North American populations of the weedy plant *Arabidopsis thaliana* is not geographically structured.. Mol Ecol.

[138] Treuren RV, Bas N, Goossens PJ, Jansen J, Van Soest LJM (2005). Genetic diversity in perennial ryegrass and white clover among old Dutch grasslands as compared to cultivars and nature reserves.. Mol Ecol.

[139] Menchari Y, Délye C, Le Corre V (2007). Genetic variation and population structure in black-grass (*Alopecurus myosuroides* Huds.), a successful, herbicide-resistant, annual grass weed of winter cereal fields.. Mol Ecol.

